# Closing the gaps: Tackling myeloma inequities in Latin America

**DOI:** 10.1016/j.htct.2025.103848

**Published:** 2025-06-05

**Authors:** Jorge Contreras

**Affiliations:** New York-Presbyterian Weill Cornell Medical Center, New York, NY, USA

Dear Editor,

Multiple myeloma (MM), a malignancy of plasma cells, poses a significant global health challenge, characterized by disparities in incidence, treatment access, and outcomes. Advances in MM therapies have significantly improved survival rates globally, but inequities in care and treatment outcomes remain particularly pronounced in Latin America.^1^, ^2^, ^3^

The global burden of MM is shaped by genetic, environmental, and demographic factors. Latin America faces higher rates of late-stage diagnoses compared to wealthier regions due to limited awareness and inadequate screening initiatives. These disparities are especially evident in Indigenous and underserved populations, reflecting systemic health inequities.^2^, ^3^, ^4^ The projected rise in MM cases highlights the urgent need for interventions to address these gaps.^4^

## Social determinants of health

Social determinants of health (SDOH) play a critical role in shaping MM outcomes. Socioeconomic status, education, and geographic location influence access to timely diagnosis and effective treatment. Patients from lower socioeconomic backgrounds are more likely to experience delayed diagnoses and poorer survival outcomes.^2^^,^^3^ Rural populations, in particular, face challenges in accessing specialists and advanced diagnostic tools, exacerbating these disparities^3^^,^^5^^,^^6^

Healthcare systems in Latin America are fragmented, leading to significant disparities between public and private sectors. Many facilities lack access to critical diagnostic tools, including next-generation sequencing and cytogenetics, which are essential for precise risk stratification. Advanced treatment options, such as autologous stem cell transplantation (ASCT) and proteasome inhibitors, remain inaccessible to many patients due to resource constraints.^3^^,^^4^^,^^5^

Financial barriers are a significant challenge for MM patients in Latin America. High costs of novel therapies combined with limited insurance coverage force many patients to opt for suboptimal care or forgo treatment altogether. Policies aimed at expanding insurance coverage and subsidizing treatment are crucial to alleviating financial toxicity.^2^^,^^6^^,^^7^

Timely initiation of treatment is a key determinant of MM prognosis. However, logistical challenges such as referral bottlenecks, lack of infrastructure, and delays in diagnosis contribute to poorer outcomes for patients in the region.^3^^,^^5^^,^^6^

MM outcomes in Latin America are poorer than in high-income countries due to delayed diagnoses, limited treatment availability, and systemic socio-economic inequities. Closing these gaps through targeted interventions, improved healthcare access, and addressing SDOH is critical to improving patient survival and quality of life.^1^^,^^2^^,^^4^

## Strategies to address disparities

Investing in healthcare infrastructure and fostering equitable access to MM therapies should be priorities for policymakers in Latin America. Collaborative efforts among governments, non-governmental organizations, and international stakeholders are essential for reducing resource gaps and promoting capacity building.^3^^,^^4^

Community engagement is vital for reducing health disparities. Awareness campaigns tailored to cultural and regional contexts can improve early detection and encourage treatment adherence. The integration of community health workers into healthcare teams has proven effective in expanding access to underserved populations.^5^, ^6^, ^7^

Expanding research initiatives in Latin America is essential for understanding region-specific challenges and developing evidence-based interventions. Establishing regional MM registries can provide valuable data on disease patterns, treatment efficacy, and healthcare disparities, enabling targeted policy decisions.^4^^,^^6^

## Conclusion

Health disparities in MM care across Latin America highlight systemic inequities rooted in socio-economic and healthcare barriers. Addressing these challenges requires a multifaceted approach involving policy reforms, community-based strategies, and enhanced research efforts. By prioritizing health equity, stakeholders can ensure that advancements in MM care benefit all patients, irrespective of their geographic or socio-economic circumstances.

## Conflicts of interest

The author declares no conflicts of interest.
